# Unraveling molecular characteristic of fluoride neurotoxicity on U87 glial-like cells: insights from transcriptomic and proteomic approach

**DOI:** 10.3389/fncel.2023.1153198

**Published:** 2023-06-09

**Authors:** Bruna Puty, Leonardo Oliveira Bittencourt, Leidiane Alencar Oliveira Lima, Jéssica Rodrigues Plaça, Aline Dionizio, Marília Afonso Rabelo Buzalaf, Bruno Duarte Gomes, Edivaldo Herculano Correa de Oliveira, Rafael Rodrigues Lima

**Affiliations:** ^1^Laboratory of Functional and Structural Biology, Institute of Biological Sciences, Federal University of Pará, Belém, Brazil; ^2^Laboratory of Cell Culture and Cytogenetics, Environmental Section, Evandro Chagas Institute, Ananindeua, Brazil; ^3^National Institute of Science and Technology in Stem Cell and Cell Therapy (INCT/CNPq) and Center for Cell-Based Therapy, Centro de Pesquisa, Inovacão e Desenvolvimento/Fundacão de Amparo á Pesuisa do Estado de São Paulo (CEPID/FAPESP), Ribeirão Preto, Brazil; ^4^Department of Biological Sciences, Bauru School of Dentistry, University of São Paulo, Bauru, Brazil; ^5^Laboratory of Neurophysiology Eduardo Oswaldo Cruz, Institute of Biological Sciences, Federal University of Pará, Belém, Brazil

**Keywords:** neurotoxicity, fluoride, proteomic, transcriptomic, glia

## Abstract

The potential of fluoride (F) as a neurotoxicant in humans is still controversial in the literature. However, recent studies have raised the debate by showing different mechanism of F-induced neurotoxicity, as oxidative stress, energy metabolism and inflammation in the central nervous system (CNS). In the present study, we investigated the mechanistic action of two F concentration (0.095 and 0.22 μg/ml) on gene and protein profile network using a human glial cell *in vitro* model over 10 days of exposure. A total of 823 genes and 2,084 genes were modulated after exposure to 0.095 and 0.22 μg/ml F, respectively. Among them, 168 were found to be modulated by both concentrations. The number of changes in protein expression induced by F were 20 and 10, respectively. Gene ontology annotations showed that the main terms were related to cellular metabolism, protein modification and cell death regulation pathways, such as the MAP kinase (MAPK) cascade, in a concentration independent manner. Proteomics confirmed the changes in energy metabolism and also provided evidence of F-induced changes in cytoskeleton components of glial cells. Our results not only reveal that F has the potential to modulate gene and protein profiles in human U87 glial-like cells overexposed to F, but also identify a possible role of this ion in cytoskeleton disorganization.

## 1. Introduction

Fluoride therapy is the main method against dental caries (Centers for Disease Control Prevention, 2001; Iheozor-Ejiofor et al., 2015), and the fluoridation of communities’ water systems is widely used due to its efficiency and cost-effectiveness (Sampaio and Levy, 2011). Since the end of the 1980s, studies report that fluoride (F) controls caries acting in the oral cavity, in contact with the teeth by favorably interfering in de- and remineralization processes, rather than acting systemically (Buzalaf and Whitford, 2011; Pessan et al., 2011). This has led to a debate about the necessity of compulsory F exposure through the water supply. This is supported by the presence of F in different sources, such as food, beverages (tea, wine and milk), infant formula, and oral care products (Levy et al., 1998, 2001, 2003; Franzman et al., 2004; Ismail and Hasson, 2008; Hujoel et al., 2009; Wong et al., 2010). The safe F dosage for humans is around 0.7 to 3 mg/day for children and adults, respectively, and the combination of different sources results in overexposure that is related to health issues (Aoun et al., 2018; Buzalaf, 2018).

Although the main side effects due to F overexposure are dental and skeletal fluorosis (Ten Cate and Buzalaf, 2019), in the last decade a few studies showed neurological damage, such as cognitive decline and lower IQ scores (Choi et al., 2012, 2015; Grandjean and Landrigan, 2014; Malin and Till, 2015). However, such results remain controversial (Duan et al., 2018; Guth et al., 2020; Seddek and Ghallab, 2020). Recently, Johnston and Strobel (2020) suggested that a positive correlation between high F levels and low IQ does not mean a causal association. In the same way, our group reported in a systematic and meta-analysis review that F exposure under therapeutical does not cause neurological damage, while studies showing overexposure to high doses showed low levels of evidence mainly due to inappropriate methodologies and high bias (Miranda et al., 2021).

While studies with F-exposed humans seem to be controversial, animal models studies suggested alterations of the central and peripheral nervous system (Niu et al., 2009; Pereira et al., 2011; Melo et al., 2017; Dionizio et al., 2018, 2020), with structural damage in areas related to motor and sensory control, such as the hippocampus, motor cortex and amygdala (Bhatnagar et al., 2002; Pan et al., 2015). *In vitro* studies reported changes in central nervous system (CNS) cells related to oxidative stress, cell death, energy metabolism and DNA damage, both at neuronal and glial cell lines in humans and rodents (Zhang et al., 2007, 2008; Shuhua et al., 2012; Qian et al., 2013; Ghasemi et al., 2018; Puty et al., 2021). These results highlight the need for further investigations aimed to understand the mode of action of F in the CNS and to identify possible molecular targets of toxicity.

Furthermore, *in vitro* and *in vivo* experiments with glial cells points to it as a possible target for F toxicity with a central role in impairments of neural development (Rasband, 2016; Jäkel and Dimou, 2017) due to the involvement of these cells in CNS homeostasis as well as in neuronal cross-talk (Aschner, 2000; Aschner et al., 2003; Sidoryk-Wegrzynowicz et al., 2011; Jäkel and Dimou, 2017). As it is well known when the CNS is a target of xenobiotics, glial cells act as the primary line of cellular defense to protect the neurons (Aschner et al., 2002; Shanker et al., 2003; Noguchi et al., 2013; Sidoryk-Wegrzynowicz and Aschner, 2013; Ishihara et al., 2019). In this scenario, glial cells could be used as indicators of CNS drug-induced toxicity with molecular changes in these cells being biomarkers to monitor CNS impairment.

Recently, our group established a single-cultured F overexposure model using two cell lines, human glial-like (U87) and neuronal-like (IMR-32) cells. Once F toxicity is dose- and time-dependent, we simulated a continuous exposure for 10 days using a low and high F concentration (0.095 and 0.22 mg/ml) based on what it is usually found in plasma samples from people living in areas of endemic fluorosis. Our results showed that only exposure to 0.22 μg/ml induced signs of toxicity in U87 glial-like cells, due to lower cell viability, changes in cell energy metabolism, decreased reduced glutathione/oxidized glutathione (GSH/GSSG) ratio, and DNA damage (Puty et al., 2021). In the present study we aimed to show the molecular targets via gene and protein modulation in the human glial-like (U87) cells using the same F concentrations described above to provide molecular insight about F toxicity on glial-like cells. For that, we examined molecular changes underlying F-exposed U87 glial cells using the global gene and protein expression profiles. The biological targets provided by gene ontology and molecular pathways were also examined to give a general overview of cell responsiveness to F toxicity.

## 2. Materials and methods

### 2.1. Cell culture and fluoride exposure

U87 glial cells (ATCC) were grown in T75 flasks containing 20 ml of Dulbecco’s Modified Eagle’s Medium supplemented with 10% of fetal bovine serum (FBS), penicillin (50 U/ml), streptomycin (25 μg/ml), gentamycin (25 μg/ml) and amphotericin B (2.5 μg/ml) at 37°C in a controlled 5% CO_2_ atmosphere. Medium was changed every 2–3 days. During experiments, cells were seeded onto 24-well plates (10,000 cells/well) and exposed to NaF (0.095 or 0.22 F μg/ml) or not, for 10 days. Throughout the 10-day exposure period, the medium from control and exposed group were replaced for a fresh one (with F or not) every 2 days. After that, the medium was completely withdrawn and the cells were detached with a solution containing 0.25% trypsin/EDTA, followed by centrifugation (3 min at 448 *g*). The medium was withdrawn from the pellet using a pipette, and pellet samples from three independently experiments (*n* = 3) were used in the subsequent assays.

### 2.2. Gene expression

#### 2.2.1. Total mRNA extraction

Total mRNA extraction was performed using the SV total RNA isolation system from Promega, according to the manufacturer’s instructions. mRNA was diluted in 15 μL of nuclease-free H_2_O. RNA quantification was performed using a Qubit 2.0 and RNA integrity was assessed by a Tapestation with High Sensitivity RNA ScreenTape (Agilent Technologies, Santa Clara, CA, USA). Only samples with RNA integrity (RIN) > 7 were used for downstream analysis.

#### 2.2.2. One-color microarray expression

The microarray gene expression assay was performed with a one-color microarrays-based gene expression analysis kit (Agilent Technologies, USA), according to the manufacturer’s instructions. In brief, total RNA from exposed and non-exposed cells were used as the template to drive cDNA synthesis with T7 RNA polymerase followed by cRNA synthesis. The cRNA was labeled with Cy3 using a Low Input Quick Amp Labeling kit (Agilent Technologies) according to manufacturer’s instructions. Labeled cRNA purification was performed using a RNeasy mini-spin kit. cRNA was quantified by spectrophotometry (ng/μL) and analyzed by the A260/280 parameter. Hybridization was performed with 300 ng of Cy3 labeled cRNA, 5 μL of 10xGene expression blocking agent and 1 μL of 25x fragmentation buffer for 17 h at 65°C and 10 rpm on a SurePrint G3 Human Gene Expression 8 × 60K microarray chip (G4851A, Agilent, USA). A microarray scanner (G4900DA, Agilent) was used with the following set-up: scan area (61 × 21.6 mm); 5 μm of resolution; green channel. Microarray scan images were obtained by Feature Extraction v10.10.

#### 2.2.3. Microarray bioinformatics analysis

Quality control and quantile normalization were performed using the limma package. Differentially expressed genes were identified based on an absolute log2 fold change level > 1 and the *p*-value adjusted by false discovery rate (FDR) *p* < 0.05. The over-representation analysis for differently expressed genes of gene ontology (GO) terms and pathways were also done with the limma package. Over-represented *p*-values were adjusted by the Bonferroni method and only adjusted *p*-values < 0.05 were considered.

### 2.3. Proteomics analysis

#### 2.3.1. Protein extraction, digestion and purification

Total protein was obtained according to a protocol published by our group (Bittencourt et al., 2017, 2019; Corrêa et al., 2020) with modifications to the cell culture samples. In brief, samples were centrifuged (3 min at 448 g, 4°C) followed by lysis buffer [7 M urea, 2 M thiourea and 40 mM dithiothreitol (DTT); diluted in ammonium bicarbonate (AmBic, 50 mM) solution] incubation with constant shaking at 4°C. Samples were then centrifuged (20,817 rpm for 30 min at 4°C) and the supernatant was collected for protein quantification by the Bradford method (Bradford, 1976). A total of 50 μg of protein was collected and the corresponding volume was completed with AmBic to reach a final volume of 50 μL (1 μg/μL). To each sample were added 10 μL of 50 mM AmBic and 25 μL of 0.2% RapiGEST™ (Waters Co., Manchester, UK) followed by 30 min incubation at 37°C. Then, 5 mM DTT was added and incubated at 37°C for 1 h, followed by incubation with 10 mM iodoacetamide for 30 min at room temperature. Protein digestion was performed with 10 μL of trypsin for 14 h at 37°C followed by 10 μL of 5% trifluoroacetic acid for 90 min at 37°C. After that, samples were centrifuged (20,817 rpm for 30 min at 6°C) the supernatants were collected and purified using C18 Spin columns (Pierce™). After purification, all samples were concentrated to an approximate concentration of 1 μg/μL and then resuspended in 12 μL of ADH (1 pmol/μL) + 108 μL of 3% acetonitrile and 0.1% formic acid for mass spectrometry analysis.

#### 2.3.2. Mass spectrometry and bioinformatic analysis

The mass spectrometry system used for the proteomic approach was a nanoAcquity UPLC-Xevo QTof MS system (Waters, Manchester, UK), using the Protein Lynx Global Server (PLGS) software, after downloading the Uniprot database. The difference in expression between the groups was analyzed by *t*-test (*p* < 0.05), using the PLGS software. After protein identification and categorization, Cytoscape 3.6.1 (Java^®^) software was used for bioinformatics analyses with the ClusterMarker plugin for protein-protein interaction networks and the ClueGO plugin for the determination of biological process groups (Bindea et al., 2009).

## 3. Results

### 3.1. Exploratory U87 transcriptomic changes under F exposure

**FIGURE 1:**
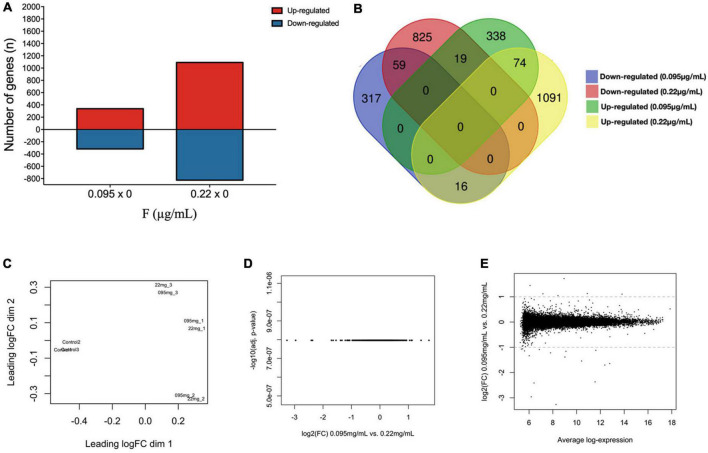
Fluoride-induced transcriptome changes in U87 glial-like cells after 10 days of exposure. Differentially expressed genes are shown as the number of genes up and down-regulated after 0.095 and 0.22 μg/ml F **(A)**. Venn’s diagram shows the overlapped gene expression when comparing 0.095 and 0.22 μg/ml **(B)**. The expression of all genes are displayed in the form of a two-dimensional PCA diagram showing sample clustering (each point represents one experiment, *n* = 3) **(C)**. Comparisons between 0.095 μg/ml and 0.22 μg/ml are shown by a volcano plot **(D)** and an MA plot **(E)**. Data are from the control group (untreated cells), 0.095 μg/ml F group and 0.22 μg/ml F group. Each dot represents a single gene. The log2 (fold change) is a measure of gene expression and the –log10 (corrected *P*-value) represents the *t*-test considering *p* < 0.05.

Supplementary Table 1 shows all differentially expressed genes (DEGs) in the comparisons 0.095 μg/ml vs. control and 0.22 μg/ml vs. control. Our results show 392 down-regulated genes and 431 up-regulated genes for 0.095 μg/ml, while 903 were down-regulated and 1,181 were up-regulated for 0.22 μg/ml (Figure 1A). Among them, 59 were down-regulated both at 0.095 μg/ml and 0.22 μg/ml while 74 genes were up-regulated at both concentrations. On the other hand, 16 genes had their expression down-regulated by 0.095 μg/ml while were up-regulated by 0.22 μg/ml and 19 genes were up-regulated by 0.095 and down-regulated by 0.22 μg/ml. A full list of overlapped genes is provided on a separate sheet in the Supplementary Table 1 and illustrated in Figure 1B. Our results also show how individual sample analysis between concentrations clustered closely using principal component analysis (PCA) (Figure 1C), which suggests no differences in gene expression between 0.095 and 0.22 μg/ml F. These results were confirmed by volcano plot (Figure 1D) and MA plot which show no DEGs in the comparison 0.095 vs. 0.22 μg/ml (Figure 1E). No significant pathways were found to be regulated by overlapped genes at both concentrations.

**FIGURE 2:**
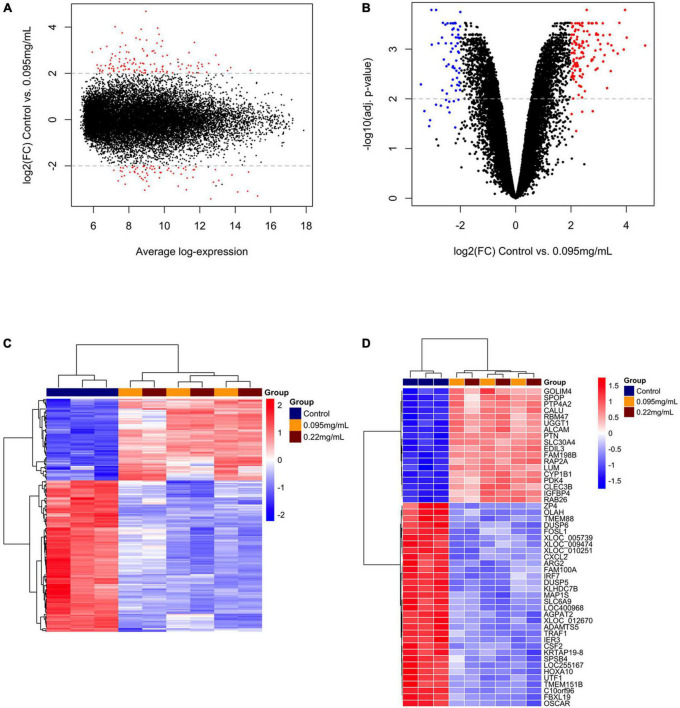
Differentially expressed genes over 10 days of 0.095 μg/ml F versus control. MA plot **(A)** and volcano plot **(B)** showing the distribution of gene expression after Benjamini-Hochberg normalization. Each dot represents a single gene. The log2 (fold change) is a measure of gene expression and the –log10 (correctedPvalue) represents the *t*-test considering *p* < 0.05. Red dots are genes that were up-regulated and blue dots genes that were down-regulated by 0.095 μg/ml F. A general overview of the differentially expressed genes is shown in the heatmap **(C)**. The top 50 genes regulated by 0.095 μg/ml F are presented **(D)**. Each square represents a single gene that is identified by a gene symbol on the right of the panel. The heatmap color code indicates up-regulation of genes compared to control (red) and down-regulation of genes compared to control (blue). The corresponding values of the heatmap are the Z-score obtained by normalizing gene expression.

**FIGURE 3:**
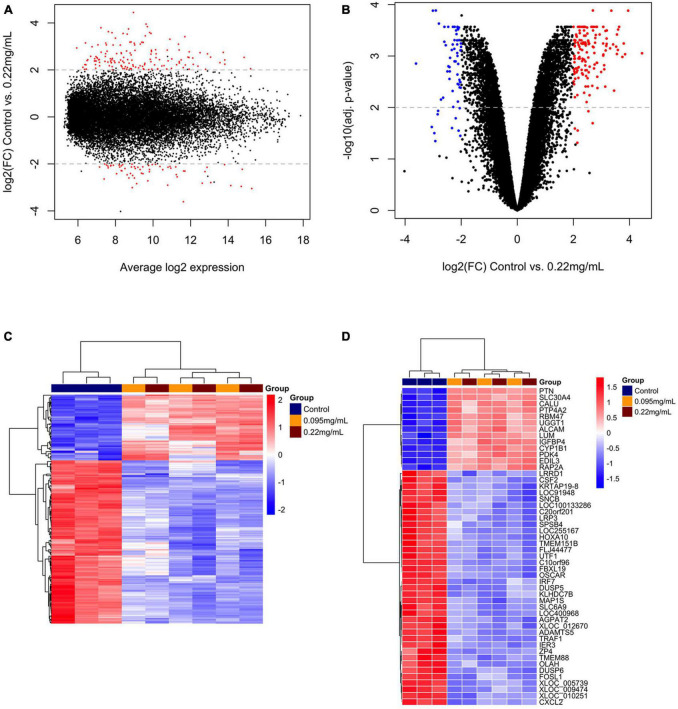
Differentially expressed genes over 10 days of 0.22 μg/ml F versus control. MA plot **(A)** and volcano plot **(B)** showing the distribution of gene expression after Benjamini-Hochberg normalization. Each dot represents a single gene. The log2 (fold change) is a measure of gene expression and the –log10 (correctedPvalue) represents the *t*-test considering *p* < 0.05. Red dots are genes that were up-regulated and blue dots genes that were down-regulated by 0.22 μg/ml F. A general overview of the differentially expressed genes is showed by the heatmap **(C)**. The top 50 genes regulated by 0.22 μg/ml F are presented **(D)**. Each square represents a single gene that is identified by a gene symbol on the right of the panel. The heatmap color code indicates up-regulation of genes compared to control (red) and down-regulation of genes compared to control (blue). The corresponding values of the heatmap are the Z-score obtained by normalizing gene expression.

A overview of the DEGs for 0.095 and 0.22 μg/ml against control is shown in Figures 2A–D, 3A–D, respectively. Figures 2A, 3A show the range of log2 fold change versus average log expression, while Figures 2B, 3B show the –log10 (adjusted *p*-value).

Figures 2C, 3C show the unsupervised hierarchical clustering in the comparison 0.095 μg/ml vs. control and 0.22 μg/ml vs. control, respectively, providing an overview of F-induced DEGs in U87 glial cells. The top 50 DEGs in the comparisons 0.095 μg/ml vs. control and 0.22 μg/ml vs. control are seen in Figures 2D, 3D, respectively.

### 3.2. Functional classification of F-induced DEGs by gene ontology

**FIGURE 4:**
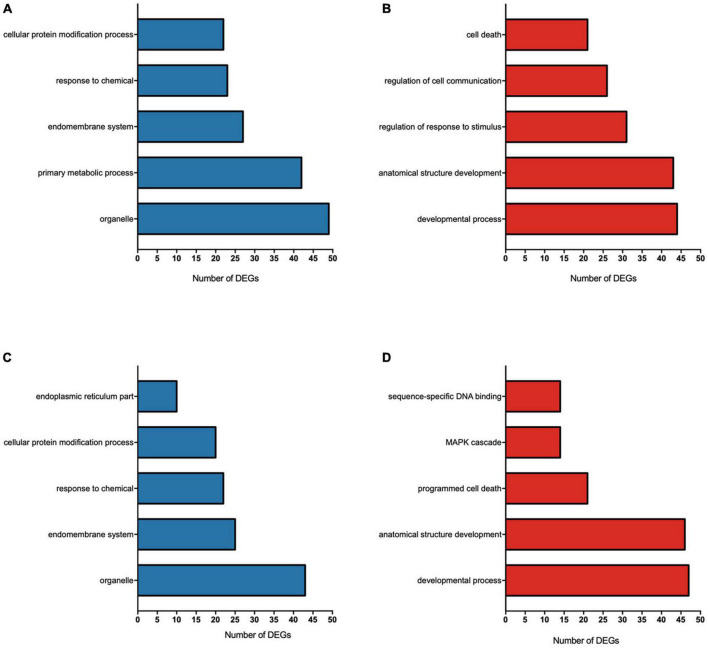
Gene ontology (GO) functional annotations for DEGs of U87 glial-like cells exposed to 0.095 μg/ml F **(A,B)** and 0.22 μg/ml F **(C,D)**. The main terms of each category affected by fluoride are represented by the number of differently expressed genes (DEGs) on each term down-regulated **(A,C)** and up-regulated **(B,D)**. The *X*-axis represents the number of DEGs and the *Y*-axis shows the GO terms of each category considering a fold change ≥ 2 and *p* < 0.05.

All GO pathways in the 0.095 μg/ml vs. control and 0.22 μg/ml vs. control comparisons are shown in Supplementary Table 2. The top 5 enriched pathways for each concentration are shown in Figures 4A–D. DEGs down-regulated by 0.095 μg/ml F led to major changes in various metabolic process pathways, the endomembrane system, the cellular response to chemical stimuli and the regulation of protein modification processes (Figure 4A), while up-regulated DEGs led to major changes in the regulation of responses to stimuli and regulation of cell death (Figure 4B). For the 0.22 μg/ml vs. control, the results indicate that down-regulated DEGs led to changes in the endomembrane system, the cellular protein modification process, the endoplasmic reticulum and response to chemical (Figure 4C), while up-regulated DEGs led to major changes in programmed cell death, sequence-specific DNA binding and MAP kinase (MAPK) cascade (Figure 4D).

### 3.3. Protein modulation by F exposure

All differentially expressed proteins (DEPs) in the 0.095 and 0.22 μg/ml groups are shown in Table 1.

**TABLE 1:** Differentially expressed proteins on U87 glial-like cells after fluoride exposure.

^a^Accession ID	Protein description	PLGS score	Fold change
**0.095 μg/ml F vs. Control**			
Q13885	Tubulin beta-2A chain	836.04	1.45
Q13509	Tubulin beta-3 chain	859.69	1.40
Q9BVA1	Tubulin beta-2B chain	836.04	1.46
A5A3E0	POTE ankyrin domain family member F	1,733.36	-0.18
P63267	Actin_ gamma-enteric smooth muscle	4,979.13	-0.18
P04075	Fructose-bisphosphate aldolase A	210.05	-0.24
P08670	Vimentin	2,784.19	-0.28
P0CG39	POTE ankyrin domain family member J	706.02	-0.30
Q9BYX7	Putative beta-actin-like protein 3	158.76	-0.39
Q562R1	Beta-actin-like protein 2	158.76	-0.41
P68133	Actin_ alpha skeletal muscle	4,979.13	-0.44
P68032	Actin_ alpha cardiac muscle 1	4,979.13	-0.44
P62736	Actin_ aortic smooth muscle	4,979.13	-0.45
P63261	Actin_ cytoplasmic 2	5,305.6	-0.47
P60709	Actin_ cytoplasmic 1	5,305.6	-0.47
Q6S8J3	POTE ankyrin domain family member E	1,736.97	-0.48
P0CG38	POTE ankyrin domain family member I	1,574.6	-0.49
Q16352	Alpha-internexin	152.62	-0.64
P07197	Neurofilament medium polypeptide	152.62	-0.68
P17661	Desmin	152.62	-0.68
**0.22 μg/ml F vs. Control**			
P07437	Tubulin beta chain	836.04	-0.12
P04350	Tubulin beta-4A chain	476.51	-0.14
P60709	Actin_ cytoplasmic 1	5,305.6	-0.63
P08670	Vimentin	2,784.19	-0.65
P17661	Desmin	152.62	-0.65
Q16352	Alpha-internexin	152.62	-0.66
P07197	Neurofilament medium polypeptide	152.62	-0.67
P63261	Actin_ cytoplasmic 2	5,305.6	-0.67
P68133	Actin_ alpha skeletal muscle	4,979.13	-0.68
P04406	Glyceraldehyde-3-phosphate dehydrogenase	143.63	-0.73
**0.22 vs. 0.095 μg/ml F**			
P0CG39	POTE ankyrin domain family member J	467.46	3.39
Q9BYX7	Putative beta-actin-like protein 3	56.42	2.80
Q562R1	Beta-actin-like protein 2	56.42	2.72
P62736	Actin_ aortic smooth muscle	772.96	2.66
A5A3E0	POTE ankyrin domain family member F	772.96	2.64
P63267	Actin_ gamma-enteric smooth muscle	772.96	2.59
P68133	Actin_ alpha skeletal muscle	772.96	2.56
P68032	Actin_ alpha cardiac muscle 1	772.96	2.56
P60709	Actin_ cytoplasmic 1	772.96	2.56
P63261	Actin_ cytoplasmic 2	772.96	2.46
Q6S8J3	POTE ankyrin domain family member E	772.96	2.34
P08670	Vimentin	1,375.87	1.82
Q13509	Tubulin beta-3 chain	733.94	-0.62

^a^Accession ID based on uniprot.org database. Values of fold change (FC) ≥ 1 represent proteins up-regulated, while FC < 1, down-regulated. Data shown proteins up- and down-regulated on 0.095 μg/ml × control, 0.22 μg/ml × control and 0.22 μg/ml × 0.095μg/ml, respectively.

Our results show that 17 proteins were down-regulated and 3 were up-regulated after exposure to 0.095 μg/ml F, while for 0.22 μg/ml F only 10 proteins were down-regulated. Comparisons between 0.22 and 0.095 μg/ml F showed that 12 proteins were up-regulated and 1 was down-regulated. Furthermore, we also identified 39 proteins with unique expression (PUE) in control when cells were exposed to 0.095 μg/ml F and 35 when cells were exposed to 0.22 μg/ml F (Table 2).

**TABLE 2:** Proteins with unique expression on control group when compared to 0.095 and 0.22 μg/ml.

Accession ID^a^	Protein description	Comparison			
		**0.095 μg/ml × C**	**PLGS score**	**0.22 μg/ml × C**	**PLGS score**
P06733	Alpha-enolase	+	309.84	+	309.84
P23528	Cofilin-1	+	795.63	+	795.63
Q9Y281	Cofilin-2	+	209.23	+	209.23
P14625	Endoplasmin	+	53.44	+	53.44
P04406	Glyceraldehyde-3-phosphate dehydrogenase	+	143.63	–	–
Q96A08	Histone H2B type 1-A	+	398.54	+	398.54
P33778	Histone H2B type 1-B	+	489.46	+	489.46
P62807	Histone H2B type 1-C/E/F/G/I	+	489.46	+	489.46
P58876	Histone H2B type 1-D	+	489.46	+	489.46
Q93079	Histone H2B type 1-H	+	489.46	+	489.46
P06899	Histone H2B type 1-J	+	489.46	+	489.46
O60814	Histone H2B type 1-K	+	489.46	+	489.46
Q99880	Histone H2B type 1-L	+	489.46	+	489.46
Q99879	Histone H2B type 1-M	+	489.46	+	489.46
Q99877	Histone H2B type 1-N	+	489.46	+	489.46
P23527	Histone H2B type 1-O	+	489.46	+	489.46
Q16778	Histone H2B type 2-E	+	489.46	+	489.46
Q5QNW6	Histone H2B type 2-F	+	489.46	+	489.46
Q8N257	Histone H2B type 3-B	+	458,83	+	458.83
P57053	Histone H2B type F-S	+	489.46	+	489.46
P00338	L-lactate dehydrogenase A chain	+	412.77	+	412.77
P41219	Peripherin	+	15.8	+	15.8
P00558	Phosphoglycerate kinase 1	+	122.95	+	122.95
P07205	Phosphoglycerate kinase 2	+	78.25	+	78.25
P07737	Profilin-1	+	209.83	+	209.83
P30613	Pyruvate kinase PKLR	+	113.4	+	113.4
P14618	Pyruvate kinase PKM	+	210.6	+	210.6
P60174	Triosephosphate isomerase	+	539.49	+	539.49
Q71U36	Tubulin alpha-1A chain	+	493.84	+	493.84
P68363	Tubulin alpha-1B chain	+	493.84	+	493.84
Q9BQE3	Tubulin alpha-1C chain	+	493.84	+	493.84
P0DPH7	Tubulin alpha-3C chain	+	670.62	+	670.62
P0DPH8	Tubulin alpha-3D chain	+	670.62	+	670.62
Q6PEY2	Tubulin alpha-3E chain	+	360.81	+	360.81
P68366	Tubulin alpha-4A chain	+	618.04	+	618.04
P04350	Tubulin beta-4A chain	+	476.51	–	–
P68371	Tubulin beta-4B chain	+	476.51	–	–
Q9BUF5	Tubulin beta-6 chain	+	39.13	+	39.13
Q3ZCM7	Tubulin beta-8 chain	+	376.95	–	–

^a^Accession ID based on uniprot.org database. Values of fold change (FC) ≥ 1 represent proteins up-regulated, while FC < 1, down-regulated.

When we compared protein expression in U87 glial-like cells in both F-exposed groups (0.22 × 0.095 μg/ml), the results showed 4 proteins that were only expressed after exposure to 0.22 μg/ml F (Table 3).

**TABLE 3:** Proteins with unique expression on 0.22 μg/ml when compared to 0.095 μg/ml.

Accession ID^a^	Protein description	PLGS score
P04406	Glyceraldehyde-3-phosphate dehydrogenase	153.64
P04350	Tubulin beta-4A chain	588.68
Q3ZCM7	Tubulin beta-8 chain	423.75
P68371	Tubulin beta-4B chain	588.68

^a^Accession ID based on uniprot.org database.

### 3.4. Functional classification by proteomics analysis

**FIGURE 5:**
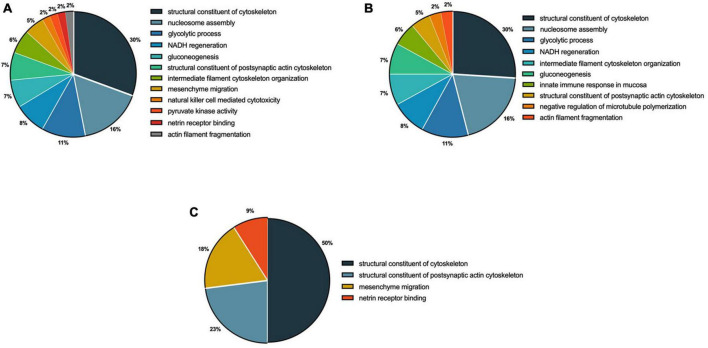
Functional distribution of proteins identified with differential expression in U87 glial-like cells exposed to fluoride. Group comparisons are shown as 0.095 μg/ml F vs. control **(A)**, 0.22 μg/ml F vs. control **(B)**, and 0.22 μg/ml F vs. 0.095 μg/ml F **(C)**. Categories of proteins are based on the gene ontology (GO) annotation of biological processes. Significant terms (kappa Score = 0.4) and the distribution according to the percentage of genes. Protein access numbers were provided by Uniprot. GO was evaluated according to the ClueGo^®^ plugin of Cytoscape^®^ software version 3.7.1.

The functional analysis based on biological processes suggested that 12 functional categories were regulated by 0.095 μg/ml F, in the following categories: structural constituents of the cytoskeleton (30%), nucleosome assembly (16%), glycolytic processes (11%), NADH regeneration (9%), gluconeogenesis (7%), structural constituents of the postsynaptic actin cytoskeleton (7%) and intermediate filament cytoskeleton organization (6%) (Figure 5A). In 0.22 μg/ml F exposed cells, the results showed regulation of 10 categories of biological processes in the following pathways: structural constituents of the cytoskeleton (26%), nucleosome assembly (20%), glycolytic processes (12%), NADH regeneration (9%), intermediate filament cytoskeleton organization (8%) and gluconeogenesis (8%) (Figure 5B). The comparison between 0.095 and 0.22 μg/ml F suggested alterations in the following four categories: structural constituents of the cytoskeleton (50%), structural constituents of the postsynaptic actin cytoskeleton (23%), mesenchymal migration (18%) and netrin receptor binding (9%) (Figure 5C).

**FIGURE 6:**
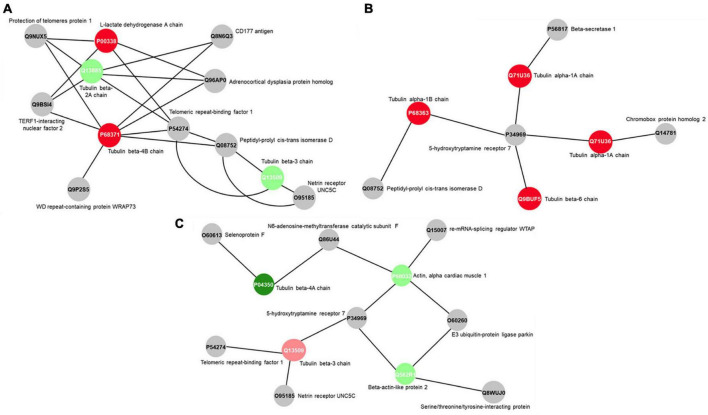
Subnetworks clustered by ClusterMarker app to determinate the interaction among identified proteins of U87 cells with differently expression on 0.095 μg/ml F vs. control **(A)**, 0.22 μg/ml F vs. control group **(B)** and 0.22 μg/ml F vs. 0.095 μg/ml F **(C)**. The node colors indicate the different status of expression of the respective protein, named by its accession ID from Uniprot.

The protein-protein interaction network (PPI) highlights, in both F exposure groups, structural proteins related to cellular cytoarchitecture. Tubulin beta-2A chain (Q13885) and tubulin beta-3 chain (Q13509) were up-regulated upon 0.095 μg/ml F exposure (Figure 6A), while tubulin alpha-1A chain (Q71U36) and tubulin alpha-1B chain (P68363) appeared only in the control group when compared to the group exposed to 0.22 μg/ml F (Figure 6B). In the 0.095 vs. 0.22 μg/ml comparison, our results showed the up-regulation of actin, alpha cardiac muscle 1 (P68032) and beta-actin-like protein 2 (Q562R1) with the higher concentration (Figure 6C). In this way, our proteomic analysis suggests an altered stability on the microtubule network of U87 glial-like cells under F exposure.

## 4. Discussion

Recent studies raised the debate about F-induced toxicity in the human CNS (Li et al., 2010; Flores-Méndez et al., 2014). Despite the lack of evidence supporting F as a potentially neurotoxic compound, studies suggested that F may be able to damage neurons, microglia, and glia (Zhang et al., 2007; Chen et al., 2017; Puty et al., 2021). Several mechanisms were pointed out as the F mode of action that results in neurotoxicity. The oxidative stress plays a role due to increasing levels of reactive oxygen species, and decreased levels of enzymatic and non-enzymatic antioxidants. Neuroinflammation, due to increasing levels of transcription factors and pro-inflammatory substances, and disorders in energy metabolism and mitochondrial dysfunction (Zhang et al., 2007, 2015; Chen et al., 2017; Yang et al., 2018). However, it is important to note that those studies often used high F concentrations which do not mimic human exposure.

We performed a large transcriptomic and proteomic analysis on a human glial-like cell model (U87) using a concentration that is similar to F plasma levels from individuals living in endemic areas for fluorosis. To our knowledge, this is the first study to investigate the effects of F in glial-like cells using omics analysis. Our results point to deep changes in the transcriptomic and proteomic profiles in a non-concentration-dependent manner, suggesting a major F effect on cell metabolism, death control, and cytoarchitecture. Several metabolic pathways, such as phosphate, macromolecule, and protein metabolic regulation were impaired, as well as proteins related to cytoarchitectures such as those belonging to tubulin and actin families.

We used F concentrations (0.095 and 0.22 μg/ml) that were close to those found in plasma samples of people living in areas of endemic fluorosis. Even when considering that the literature showed wide variation in human plasma F levels (0.017 to 1.43 μg/ml), depending on factors such as geographical region, cultural habits, and health status (Sener et al., 2007; Rafique et al., 2012; Fernando et al., 2020), it is important to highlight that the concentration of 0.095 μg/ml F is most likely to be observed in human plasma samples based on an animal model of F toxicity (Pereira et al., 2016; Dionizio et al., 2018; Miranda et al., 2018). In fact, our group has previously shown that plasma levels around 0.095 and 0.22 μg/ml, in animal models, are related to changes in the central nervous system. In this study, animals were exposed to 50 μg/ml F for 15 days and presented plasma F levels close to the concentration of 0.095 μg/ml triggering oxidative stress and proteomic modulation of the hippocampus (Ferreira et al., 2021). In previous work, we showed the F harm potential on U87 glial cells after 3, 5, and 10 days of exposure using 0.095 and 0.22 μg/ml. Signs of F toxicity were seen only after exposure to 0.22 μg/ml regarding a small decrease in cell viability, changes in energy metabolism, and cell cytoarchitecture (see Puty et al., 2021).

In the global microarray and protein profile analysis performed here, our results revealed that both 0.095 and 0.22 μg/ml F were able to modulate gene and protein networks of U87 glial-like cells. The functional classification of differentially expressed genes modulated by F exposure revealed similarities in the 0.095 and 0.22 μg/ml concentrations, suggesting molecular toxicity via regulation of several pathways related to cell death and metabolism control. The down-regulation of the endomembrane system was the top functional classification, according to GO annotation, modulated by F in a concentration-independent manner. The endomembrane system is a system derived from the endoplasmic reticulum that allows cell compartmentalization and a high degree of cell specialization and it is suggested to be modulated by F in different models of F toxicity (Arensdorf et al., 2013; Tabuchi et al., 2014; Søreng et al., 2018). Our data also showed that both F concentrations were able to modulate the MAPK, ERK1, and ERK2 cascades. These metabolic pathways are related to cellular proliferation/protection response, as well as cell death induction. In addition, the functional classification also identified pathways related to the cellular response to chemical stimuli, suggesting an adaptation mechanism to control F-induced stress over 10 days of F exposure.

MAPK plays an important role in F toxicity in different models, such as silkworm and ameloblast cells (Suzuki et al., 2015; Liu et al., 2021). MAPK up-regulation is strongly associated with different kinds of stress responses, and both MAPK and ERK are activated during the endoplasmic reticulum (ER) stress response (Hung et al., 2004; Li and Holbrook, 2004; Darling and Cook, 2014). The cell death initiated by ER stress after F exposure was demonstrated for different organisms (Kubota et al., 2005; Sharma et al., 2008; Wei et al., 2013; Zhou et al., 2013; Tabuchi et al., 2014) and may be induced by the activating transcription factor (ATF) family (Edagawa et al., 2014). Our data point to a significative modulation of different genes in the ATF4 pathway, as well as genes that are considered to be ATF4 targets. In this way, since ATF4 is involved in several classes of biological function, such as the biosynthesis, folding, and assembly of proteins, metabolism, oxidative stress, and apoptosis, we conclude that there is an important role of the ATF4 in the F exposure mode of action. The ATF4-target genes DDIT3, IL-8, IGFBP-1, ATF-3, and CEBPB were up-regulated after F exposure. The DDIT3 gene was modulated under F exposure on epithelial rat cells (ROE2) as an indicator of cell damage and is considered to be a key regulator of cell death mediated by ER stress (Tabuchi et al., 2014). Studies found that ROE2 cells exposed to F were modulated for *EGR1*, *FOS*, and *IL6*, which were up-regulated in the present study (Zinszner et al., 1998; Tabuchi et al., 2014). The regulation of those genes may be related to pro-cell death pathways, suggesting that concentrations such as the ones used in our study may be potentially harmful to U87 cells.

Another important result of the modulation of the ATF4 pathway was the up-regulation of TRIB1, CHAC1, and SESN2. The over-expression of CHAC1 and SESN2 is associated with human GSH depletion and changes in cell metabolism (Crawford et al., 2015; Garaeva et al., 2016). As we showed in a previous study, F leads to a decrease in ATP levels and cell death without activation of pro-apoptotic death as caspase3/7 (see Puty et al., 2021). We then conjecture that even the F concentrations used here may lead to ER disturbances mediated by the ATF4 pathway, causing physiological stress mediated by decreased levels of ATP and GSH/GSSG, and cell death.

The activation of the TRIB1 gene was associated with a reduction in protein synthesis (Jiang and Wek, 2005; Soubeyrand et al., 2016). Interestingly, our functional analysis revealed a wide modulation of mechanisms for protein synthesis/modification related to the extracellular matrix (see Figure 4). In order to better understand the changes in the protein profile we performed a global proteomic analysis. We identified only 17 up-regulated and 4 down-regulated proteins in 0.095 μg/ml vs. control and only 10 down-regulated proteins in 0.22 μg/ml vs. control. This poor correlation of transcriptomic and proteomic data may be attributed to posttranslational changes in the control of gene expression and the reduction of protein synthesis mediated by ATF4. However, we point out the importance of combining these two methods to investigate the effect of gene/protein changes in cellular and molecular processes after xenobiotics exposure (Udayan and Harihara, 2005). Although the regulation of some proteins was found to be different at both concentrations vs. control, the cellular behavior after F exposure seemed to be similar. We showed two main processes impaired by F exposure on the proteomic analysis: (1) cellular metabolism, via glycolytic processes, NADH regeneration, and gluconeogenesis, and (2) cellular cytoarchitecture, via structural constituents, and the organization of the cytoskeleton and nucleosome assembly. Despite the lack of an exact correlation between genes and protein modulation, the biological functional domains and their corresponding modulated pathways were the same.

Impaired energy metabolism induced by F was reported in a rodent model (Barbier et al., 2010; Pereira et al., 2018; Zuo et al., 2018). Recently, Araujo et al. (2019) showed changes in proteins related to glycolysis and gluconeogenesis in the mitochondria of rats exposed to F concentrations similar to the ones we used [50 μg/ml–since rats metabolize F 5–10 times faster than humans (Dunipace et al., 1995)]. The proposed mechanism of mitochondria impairment follows the decreased expression of fructose-biphosphate aldolase A and glyceraldehyde-3-phosphate dehydrogenase, both down-regulated in the present study, Table 1 (P0475- fructose-biphosphate aldolase A and P04406- glyceraldehyde-3-phosphate dehydrogenase).

We demonstrated alterations in the main cytoskeleton constituents that did not depend on F concentration. Despite the evidence in literature has suggested that the cytoskeleton alterations after F exposure occur in an indirect way via oxidative stress and metabolic changes (Wilson and González-Billault, 2015; Zepeta-Flores et al., 2018), our results point to a direct effect. In a previous study of our group, we showed that at the lower concentration used in the present study (0.095 μg/ml), there was no change in the oxidative stress parameters such as GSH, ROS, lipidic peroxidation and DNA integrity, and levels of cellular ATP (Puty et al., 2021).

The tubulin family is composed of α-, β- and γ-tubulins, and assembly and disassembly changes may have an impact on the morphological organization, cell migration, vesicle trafficking, cell compartmentalization, and lead cells to death (Lepekhin et al., 2001; Hohmann and Dehghani, 2019). The actin isoforms were modulated at 0.095 and 0.22 μg/ml F. Both concentrations led to the downregulation in the following actin isoforms: alpha isoforms (P68133; P68032; P62736), cytoplasmic-2 (P63261) and cytoplasmic 1 (P60709), beta isoforms (Q562R1; Q9BYX7) and gamma isoforms (P63267). However, regarding tubulin, our results pointed to strong differences in the impact of F exposure. While β-tubulins, 2A, 2B, and 3 (Q13885, Q9BVA1, Q13509, respectively) were up-regulated by 0.095 μg/ml, we observed a down-regulation on tubulin beta chain (P07437) and tubulin beta-4A chain (P04350) after 0.22 μg/ml. We also observed different classes of tubulin being expressed only in the control group compared to 0.095 μg/ml, such as tubulin alpha-1B chain (P68363), tubulin alpha-4A chain (P68366), tubulin alpha-3C chain (P0DPH7) and others (see Table 2). The tubulin beta chains 4A, 4B and 8 (P04350, P68371, Q3ZCM7, respectively) for 0.22 vs. 0.095 μg/ml were only expressed at the higher concentration (dramatically reduced at the lower concentration), and the tubulin beta 3 chain (Q13509) was down-regulated at 0.22 μg/ml. There is evidence showing a relationship between increasing beta tubulin levels and subsequent cell death (Weinstein and Solomon, 1992; Alvarez et al., 1998), which could be minimized by the co-overexpression of alpha-tubulin (Weinstein and Solomon, 1992; Alvarez et al., 1998). Besides the widely known effects of microtubule damage on the cell cycle and morphology, our findings suggest that the fluctuation of actin and tubulin expression under F exposure may trigger cell death by microtubule disassembly because the higher F concentration is, the lower the regulation of alpha subunits and higher beta subunits. Those results are in agreement with our previous work where we verified significant spreading and cell density changes for 0.22 μg/ml F followed by increased DNA fragmentation (see Puty et al., 2021). The dynamics can be observed in Figures 5, 6, where interactions in tubulin subunits were observed in two PPI.

In conclusion, our study provides valuable insights about the mode of action of F in U87 glial-like cells covering potential molecular alterations, corroborating the hypothesis of the F impact on cellular energy metabolism. We also showed F-mediated modulation of actin filament constituents, such as cytoskeleton and tubulin, suggesting an emerging target of F toxicity. Since our data were collected after a chronic model of F exposure it is important to point out that the overall gene and protein expression alteration may represent both F induced gene/proteins expression as well as adaptive changes after exposure. Finally, our results indicate that fluoride-induced damage on glial cells may happen only at overexposure to high levels of fluoride while there is no evidence of safety issues related to the optimal fluoride levels.

## Data availability statement

The datasets presented in this study can be found in online repositories. The names of the repository/repositories and accession number(s) can be found in the article/Supplementary material.

## Ethics statement

Ethical review and approval was not required for the study in accordance with the local legislation and institutional requirements.

## Author contributions

BP, EO, and RL: conceptualization. BP, AD, and RL: methodology. BP, LB, and LL: formal analysis. BP and EO: investigation. EO, RL, and MB: resources. BP, JP, AD, and BG: data curation. BP, RL, MB, and EO: writing. RL, MB, EO, and BG: writing—review and editing. All authors contributed to the article and approved the submitted version.
